# Characterization of doxycycline-dependent inducible Simian Virus 40 large T antigen immortalized human conjunctival epithelial cell line

**DOI:** 10.1371/journal.pone.0222454

**Published:** 2019-09-11

**Authors:** Arisa Mitani, Takeshi Kobayashi, Yasuhito Hayashi, Natsuki Matsushita, Sachi Matsushita, Saori Nakao, Naoko Takahira, Atsushi Shiraishi, Yuichi Ohashi

**Affiliations:** 1 Department of Ophthalmology, Ehime University Graduate School of Medicine, Shitsukawa, Toon, Ehime, Japan; 2 Department of Ophthalmology and Regenerative Medicine, Ehime University Graduate School of Medicine, Toon, Ehime, Japan; 3 Division of Laboratory Animal Research, Aichi Medical University, Nagakute, Aichi, Japan; 4 Translational Research Center, Ehime University Hospital, Toon, Ehime, Japan; 5 Department of Biochemistry, Aichi Gakuin University School of Dentistry, Nagoya, Japan; Cedars-Sinai Medical Center, UNITED STATES

## Abstract

**Purpose:**

To present the properties of a newly developed immortalized human conjunctival epithelial cell (iHCjEC) line.

**Methods:**

iHCjECs were developed to induce Simian Virus 40 large T-antigen (SV40LT) by incorporating lentivirus in a tetracycline (Tet)-regulated gene-expression system into primary cultures of human conjunctival epithelial cells. The population doubling time and morphology of the iHCjECs were analyzed. The expressions of CK13, CK19, CK12, and MUC1, MUC4, MUC16, and MUC5AC were determined by real time PCR and immunohistochemically under different culture conditions. The organotypic culture model in which iHCjECs were cultured on rabbit conjunctival fibroblast-embedded collagen gel was used to characterize the iHCjECs.

**Results:**

The iHCjECs cultured with doxycycline (Dox) continued to proliferate for at least 20 passages and had a cobblestone-like appearance. The expressions of CK13 and CK19 but not CK12 were detected in the iHCjECs, and the expression of CK13 increased in culture media lacking Dox (Dox-). The expressions of MUC1, MUC4, MUC16, and MUC5AC were detected in iHCjECs, and a relatively strong immunostaining of MUC5AC was detected with Dox(-) added 5% FBS. Stratified iHCjECs were observed in organotypic culture at 5 days.

**Conclusion:**

The iHCjECs had high proliferation rates and abilities to control the differentiation potency to control the expression of SV40 LT-antigen with Tet-regulated gene-expression system. They are able to express the mucin gene repertoire of their native epithelia. The iHCjECs can be a useful experimental cell line to study conjunctival epithelial cell characteristics and for pathophysiological and toxicological studies.

## Introduction

The surface of the eye is exposed to the outside world and is subject to infections, drying, and injury. The conjunctival epithelial cells on the surface protect the eye by maintaining a healthy ocular surface. The conjunctival apical epithelial cells express membrane-associated mucins, and the conjunctival goblet cells secrete mucins to protect and maintain the hydration of the ocular surface [1,2]. It is important to obtain more information on the physiology of the conjunctival epithelial cells to gain a better understanding of the ocular surface homeostasis.

There are several ways to obtain human conjunctival epithelial cells for investigations on the physiology of the surface of the eye. Human biopsy specimens and impression cytology can provide human conjunctival epithelial cells, however the sample size is limited and normal human tissue is not always available. In vitro cell culture systems offer a possibility of studying the effects of metabolites, mediators, and drugs on the behavior of living cells in a controlled environment. Primary cultures of human conjunctival epithelial cells have been shown to have the ability to produce and secrete mucin-type glycoproteins [3,4,5]. However, these primary cultures are prepared from human conjunctiva biopsy specimens, thus the tissue availability is limited and the amount and longevity of the cells are limited.

Immortalized conjunctival epithelial cell lines have been established and used for investigations. The Wong-Kilbourne derivative of Chang cells [6] (American Type Culture Collection [CCL] 20.2 clone 1-5c-4; Manassas, VA) is listed as being conjunctival in origin [7], Gipson et al have developed a human conjunctival epithelial cell line [8] and O’Sullivan et al have developed an immortalized rat conjunctival epithelial cell line [9].

The development of techniques to immortalize epithelial cells by preventing telomere shortening by transduction with hTERT, the catalytic subunit of the telomerase holoenzyme, was originally purported to confer replicative immortality without loss of differentiation potential [10,11]. During the course of the development of different cell lines, it became apparent that hTERT transduction was not sufficient to immortalize all cell types including keratinocytes [11]. On the other hands, it is known that immortalization with viral oncogenes, such as the SV40 large T (SV40LT)-antigen, has high proliferative ability, and immortalized cell lines developed by viral oncogenes often lose the characteristics of the original cell types because of a disruption of the natural cell differentiation programs [12]. In the tetracycline (Tet)-regulated gene-expression systems [13], the transcriptional regulation of target gene expression relies on the activity of a transregulatory protein that can be activated or repressed by tetracycline or its analog doxycycline (Dox).

The purpose of this study was to develop an immortalized human conjunctival epithelial cell (iHCjEC) line that has a doxycycline-dependent inducible SV-40LT antigen followed by transduction with hTERT. The iHCjECs were able to control their rate of replication and differentiation, and their ease of handling and maintenance of their conjunctival characteristics made them suitable for experimental use. We report that the iHCjECs continued to replicate in culture media containing Dox, and that iHCjECs expressed the mucin and keratin genes repertoire that their native conjunctival epithelia produce. These iHCjECs can be used to study the physiopathology of human conjunctival cells.

## Materials and methods

### Isolation of human conjunctival epithelial cells

The conjunctival tissues were collected from the inferior fornix region of patients undergoing conjunctivochalasis surgery at the Ehime University Hospital. An Informed consent was obtained from the patients, and the procedures were approved by the Institutional Review Board of the Ehime University Graduate School of Medicine (1407002, and 1805007). In addition, the procedures conformed to the Declaration of Helsinki.

The removed tissues were incubated in 250 U/ml dispase II (Roche Diagnostics, Basel, Switzerland) in DMEM (Invitrogen, Tokyo, Japan) at 4°C for 5 hours. The conjunctival epithelial cells were carefully scraped from the specimen with scalpels while viewing the tissue under a stereoscopic microscope. The cells were dissociated into single cells by exposing them to TrypLE™ Express Cell Dissociation Enzyme solution (Life Technologies, Carlsbad, CA).

### Construction of plasmid DNA and preparation of lentivirus vector

**Fig 1:**
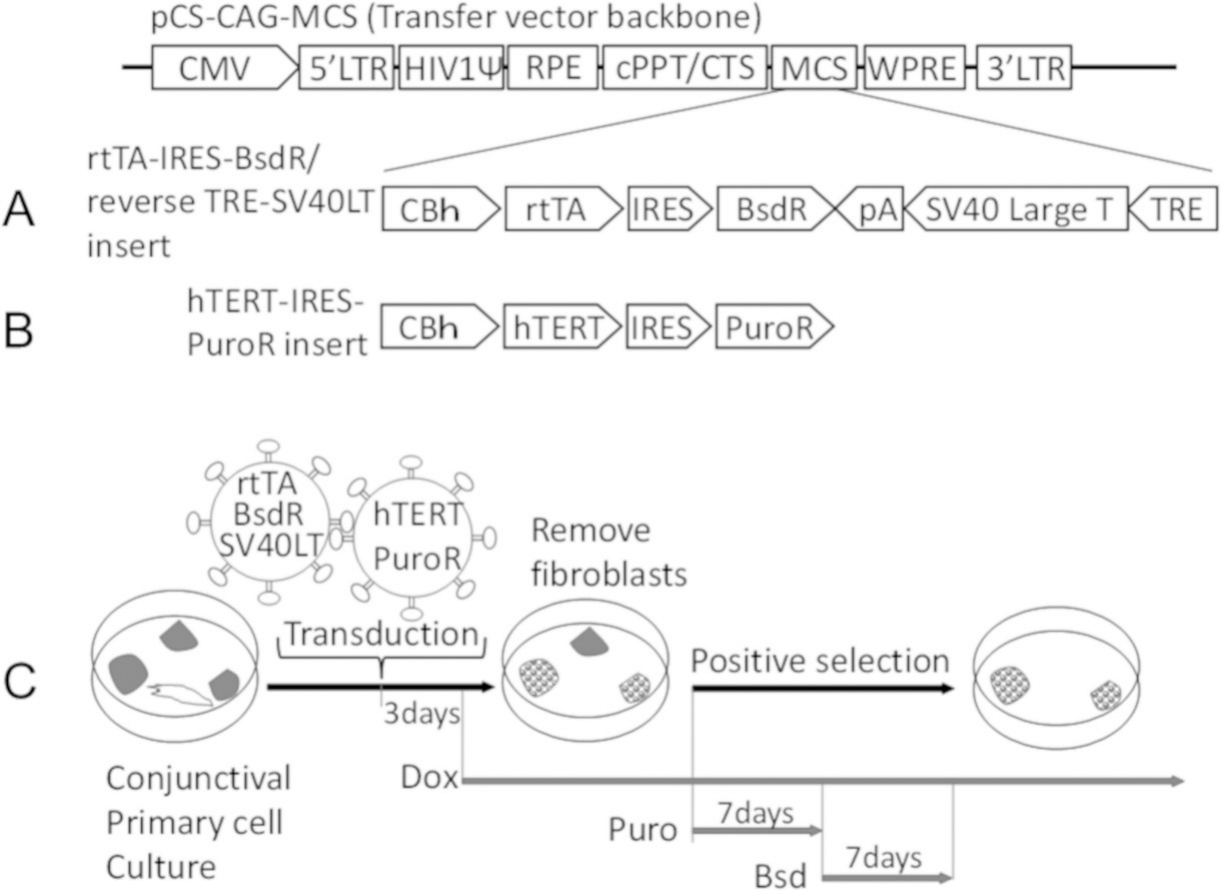
Structure of transgene in a single lentiviral vector and schematic representation of cultures for immortalized human conjunctival epithelial cells (iHCjECs). Schematic representation of transgene cassettes (A, SV40LT antigen gene; B, hTERT gene) inserted into an HIV-based self-inactivating lentiviral expression vector. A: The transgene cassette of SV40LT antigen gene consists of two distinct transcription units; an expression unit of reverse tetracycline-controlled transactivator (rtTA) under the control of the universal CBh promoter, and another containing SV40LT antigen gene under the control of a Tet-responsive element (TRE) promoter. B: To construct the lentiviral expression plasmid for the continuous expression of hTERT, the hTERT sequence was inserted downstream of CBh. C: To immortalize the human conjunctival epithelial cells, the cells were suspended with HEK293T reporter cells with lentiviral vectors and cultured on plastic plates coated with type I collagen at 37°C with 5% CO_2_. To select the transfected cells, Puromycin (1 μg/ml) was added at 80% confluency and cultured for 1 week. After selection with Puro, Bsd (25 μg/ml) and Dox (1 μM) were added and cultured for 1 week. After selection by Bsd, the cells were cultured with CnT-Prime 2D Diff containing Dox (1 μM). The medium was changed every 2 to 3 days and subcultured when the cells were subconfluent.

A human immunodeficiency virus (HIV)-based self-inactivating lentiviral expression plasmid (pCS-CAG-MCS) and two packaging plasmids, pCAG-HIVgp and pCMV-VSV-G-RSV-Rev), were provided by RIKEN BioResource Center, Tsukuda, Japan. These plasmids were used for the preparation of lentiviral vectors [14]. The coding sequence of the SV40LT and hTERT were obtained from Addgene (Watertown, MA); pLenti CMV/TO small + large T antigen (w611-7), and hTERT gene pLOX-TERT-iresTK. The construction of the doxycycline-dependent inducible EGFP, pCS-CAG-rtTA-IRES-Bsd-reverse EGFP-reverse TRE, has been described in detail [13]. To construct the doxycycline-dependent inducible SV40LT, the CAG promoter and EGFP were replaced by the CBh promoter and SV40LT fragment, respectively (Fig 1A). To construct the lentiviral expression plasmids for continuous expression of hTERT, the CAG promoter sequence of pCS-CAG-MCS was replaced by the CBh promoter, yielding pCS-CBh-MCS. Then, the DNA fragment of IRES-Puro was inserted into muticloning sites (MCS) of pCS-CBh-MCS. Finally, the hTERT sequence was inserted downstream of the CBh promoter (Fig 1B). All cloned sequences were confirmed by an automatic sequencing machine (3500 Genetic Analyzer, Applied Biosystems™, Tokyo, Japan).

HEK293T cells were obtained from the RIKEN BioResourse Center and maintained in Dulbecco’s Modified Eagle Medium (Life Technologies) supplemented with 10% fetal bovine serum, 1% L-glutamine, 110 mg/L of sodium pyruvate, 100 units/mL of penicillin, and 100 μg/mL of streptomycin (Life Technologies). The HEK293T cells were used to prepare lentiviral vector particles which were generated by standard transfection procedures.

### Primary cultures and immortalization of human conjunctival epithelial cells

The culture media, CnT-Prime and CnT-Prime 2D Diff, were purchased from CELLnTEC (Bern, Switzerland). Streptomycin, penicillin, amphotericin B mixture (Anti-Anti), doxycycline (Dox), and TrypLE^TM^ Express were obtained from Life Technologies. Puromycin dihydrochloride (Puro) was purchased from Sigma-Aldrich (St. Louis, MO), and Blasticidin (Bsd) was purchased from Nacalai Tesque (Kyoto, Japan).

Normal primary human conjunctival epithelial cells were cultured on plastic plates coated with type I collagen (IWAKI, Shizuoka, Japan) in CnT-Prime with 1% Anti-Anti and Dox (1 μM) with 5% CO_2_ at 37°C. The medium was changed every 1 to 2 days, and cell replication was assessed by counting the number of cells under a phase-contrast microscope.

To immortalize the human conjunctival epithelial cells (iHCjECs), the cells were suspended in a virus solution (1.5 × 10^6^ TU/100 μl) containing 1 ml of CnT-Prime and 1% Anti-Anti and cultured on plastic plates coated with type I collagen at 37°C with 5% CO_2_. After 3 days, the medium was changed to CnT-Prime containing 1% Anti-Anti and Dox (1 μM) and cultured until they were subconfluent and then subcultured. To remove the contaminating fibroblasts, the cells were exposed to 1 ml of TrypLE^TM^ Express at 37°C for 8 minutes, and the detached cells which can be considered to contain fibroblasts due to their higher susceptibility to trypsin than epithelial cells, were removed. To select the transfected cells, 1 μg/ml of Puro was added to the subconfluent culture for 1 week. After selection with Puro, the medium was replaced with CnT-Prime 2D Diff containing 1% Anti-Anti, Bsd (25 μg/ml), and Dox (1 μM) and cultured for 1 week (Fig 1C). After selection by Bsd, the cells were transferred to a 100 mm collagen-coated culture dish and cultured with CnT-Prime 2D Diff containing 1 μM Dox. The medium was changed every 2 to 3 days. The subconfluent cultures were subcultured at a density of 1 x 10^4^ cells/ml.

For all studies of the expression of cytokeratins and mucins, 12th passaged iHCjECs were plated at a density of 1.0 X 10⁴ cells/cm^2^ on plastic plates coated with type I collagen (IWAKI). They were cultured in CnT-2D-Diff with Dox (1 μM) at 37°C with 5% CO_2_. After 3 days, the culture medium was replaced with fresh medium with or without Dox or with 5% FBS-containing medium without Dox and cultured for 5 additional days.

### Organotypic culture of iHCjECs on conjunctival fibroblast-embedded collagen gel

To isolate rabbit conjunctival fibroblasts (RCjFs), Japanese white rabbits weighing 2–3 kg were purchased from the Kitayama Labes Co. (Kyoto, Japan). The rabbits were handled in accordance with the guidelines of the ARVO Statement for the Use of Animals in Ophthalmic and Vision Research. The animals were euthanized with an overdose of pentobarbital, and the conjunctival tissues were excised and washed in PBS(-). The tissues were incubated in 250 U/ml of dispase II in DMEM at 4°C for 5 hours. The epithelial conjunctival connective tissues were removed with scalpels, and the remaining stromal tissue was cut into 2 mm x 2 mm pieces and placed on 10 cm culture dishes (Asahi Techno Glass, Funabashi, Japan). The tissues were cultured in DMEM supplemented with 10% FBS and 1% antibiotic antimycotic solution at 37°C under 95% humidity and 5% CO_2_. The subconfluent rabbit conjunctival fibroblasts (RCjFs) were subcultured at a density of 5 x 10^3^ cells/cm^2^ and twice passaged RCjFs were used for the experiments.

RCjFs embedded collagen gels were prepared as described in detail [15]. Briefly, the collagen gel was prepared by mixing six volumes of ice-cold porcine collagen type I solution with one volume of 8 x DMEM, ten volumes of 1 x DMEM supplemented with 20% FBS, and one volume of 0.1 N NaOH (collagen solution). Then, 1 ml of this solution was added to each 24 mm diameter six-well culture plates with a 3 mm-pore membrane (Corning Coster Corporation, Cambridge, MA). Following polymerization of the gel in the inserts at 37°C, two volumes of RCjF suspension (5 x 10^5^ cells/ml) in 1 x DMEM supplemented with 10% FBS were added to eight volumes of the collagen solution until the final collagen concentration was 0.8 mg/ml. Then, 3.5 ml of the fibroblast-containing collagen solution was added to each insert. When the fibroblast-containing gel polymerized, DMEM supplemented with 10% FBS and ascorbic acid (final concentration: 50 ng/ml) was added. The gel was kept submerged in the media for five days until the fibroblasts contracted into the collagen gel.

The iHCjE cells (12th passage) were seeded at a density of 1.0 x 10⁴ cells/cm^2^ on the RCjF-embedded collagen gel and cultured in DMEM/Ham’s F-12 with or without Dox (1 μM) or in 5% FBS containing medium without Dox at 37°C with 5% CO_2_ for 5 days to achieve epithelial stratification.

### Western immunoblot analysis

**Fig 2:**
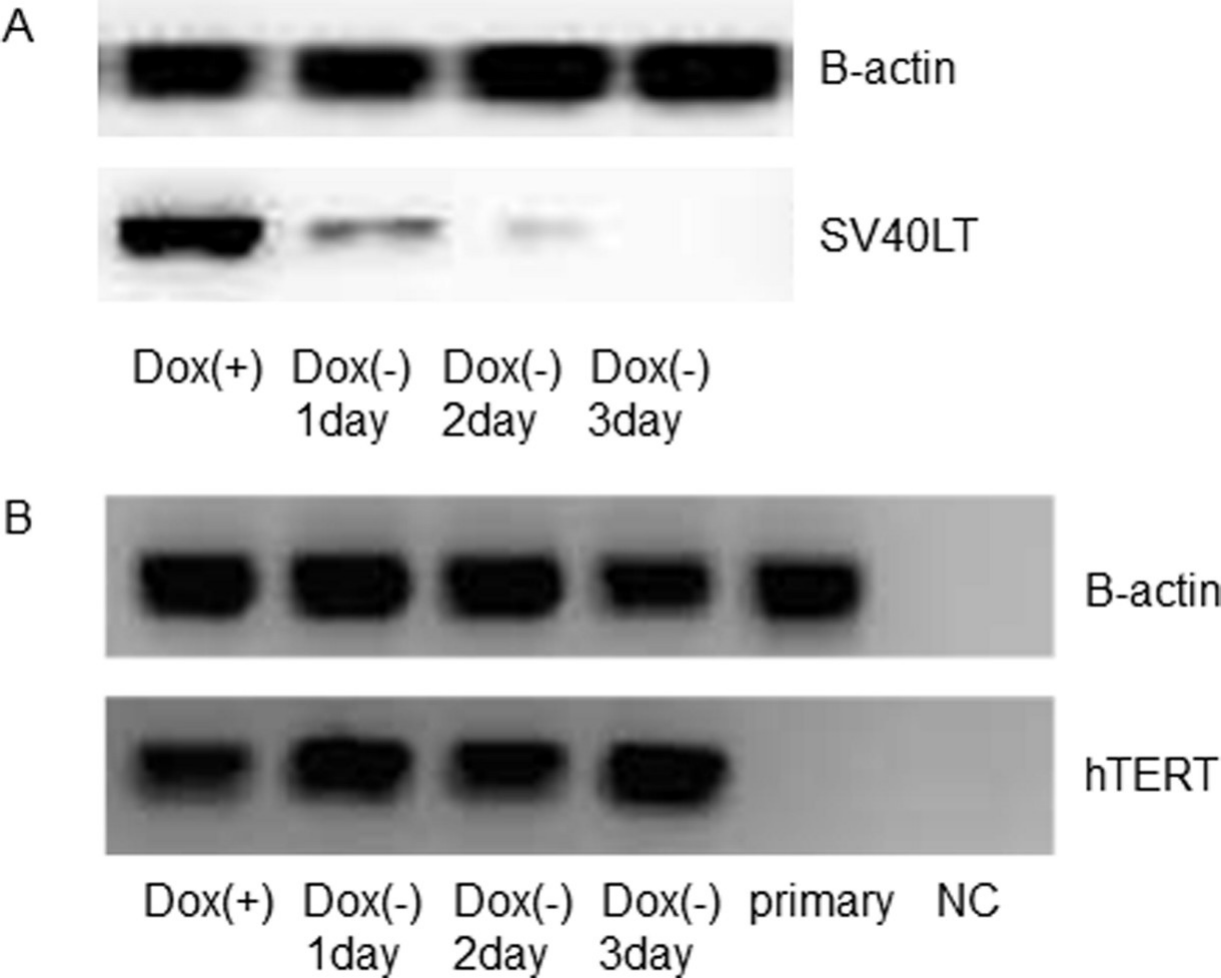
Western blot analysis of SV40LT expression and PCR of hTERT expression in iHCjECs cultured with Dox or without Dox. A: The expression of SV40LT antigen was observed in cells cultured in a medium containing Dox, and the expression gradually decreased at 24 h, 48 h, and 72 h after exchanging the medium to one without Dox. No expression of SV40LT antigen was observed in cells cultured for 72 hours in a medium without Dox. B: Expression of hTERT gene is observed in cultured cells either with or without Dox. NC: negative control.

The iHCjE cells (12th passage) were plated at a density of 1.0 X 10⁴ cells/cm^2^ on plastic plates coated with type I collagen in CnT-2D-Diff with Dox (1 μM) at 37°C in 5% CO_2_ for 3 days and cultured to subconfluence (Dox+). Then, the culture medium was exchanged with fresh medium without Dox for 1, 2, or 3 days. The proteins were collected every day for 3 days (Dox-1 day, -2 days, and -3 days; Fig 2A).

The protein was extracted from the cell cultures with a radioimmunoprecipitation (RIPA) buffer (150 mM NaCl, 50 mM Tris, pH 8.0, 1% NP-40, 0.5% deoxycholate, 0.1% SDS; Wako, Osaka, Japan) supplemented with a cocktail of protease inhibitors (Halt^TM^ Protease Inhibitor Single-Use Cocktail; Thermo Scientific, Tokyo, Japan). After homogenization with a pellet pestle, the cell lysates were centrifuged at 15,000 rpm for 10 minutes, and the protein concentration of the supernatant was determined with the Pierce BCA Protein Assay Kit (Thermo Scientific). For the analysis of the SV40LT antigen, proteins were separated on a 5% to 20% gradient SDS-polyacrylamide gel (Super Sep^TM^ Ace; Wako; Japan) and transferred to a PVDF membrane (Immun-Blot PVDF Membrane for Protein Blotting; Bio-Rad, Hercules, CA). The membrane was then blocked with 5% nonfat milk in 0.02M Tris-HCL buffer pH: 7.4 / 0.15M NaCl (TBS), containing 0.05% Tween 20 (TBS-T), incubated overnight with mouse anti-SV40LT antibody (1:500, Pab101, Santa Cruz Biotechnology, Texas), or mouse anti-β-Actin antibody (1:5,000, clone A5441, Sigma, St. Louis, MO) at 4°C. The membrane was washed, incubated with horseradish peroxidase-conjugated anti-mouse IgG (1:1,000; VECTOR Laboratories, Burlingame, CA) at room temperature for 1 hour and then washed again. A chemiluminescent reagent (Amersham^TM^ ECL^TM^ Prime Western Blotting Detection Reagent; GE Healthcare, Little Chalfont, United Kingdom) was applied to the membrane and the luminescent signal was detected by a Molecular Imager ChemiDoc^TM^ XRS System (Bio-Rad).

### Growth curve of iHCjECs

iHCjE cells (12th passage) were placed into 100 cm^2^ tissue culture flasks (Corning,Corning, NY) at a seeding density of 1.0 X 10⁴ cells/cm^2^ in CnT-2D-Diff with Dox (1 μM) and cultured for 24 hours at 37°C in 5% CO_2_. Then, the culture medium was exchanged with fresh medium with or without Dox and cultured for another 24 h, 48 h, or 72 h. The number of cells harvested by trypsinization was determined by a cell counter (Luna automated cell counter; Logos Biosystems, Gyunggi, Korea). The growth curve was obtained by plotting the logarithm of the cell number against the growth time.

### Analyses of gene expression

The iHCjE cells (12th passage) were plated at a density of 1.0 X 10⁴ cells/cm^2^ on plastic plates coated with type I collagen and cultured in CnT-2D-Diff with 1 μM Dox at 37°C with 5% CO_2_ for 3 days until the cells were subconfluent (Dox+). After that, the culture medium was exchanged with fresh medium without Dox (Dox-) for 1, 2, or 3 days. Total RNA was extracted from the iHCjECs with RNeasy Mini Kit (Qiagen Japan, Tokyo, Japan) according to the manufacturer's protocols. SuperScript VILO cDNA Synthesis Kit (Invitrogen) was used to convert the total RNA to cDNAs by reverse transcription according to the manufacturer's protocol.

Polymerase chain reaction (PCR) was performed with Premix Taq (Takara, Ohtsu, Japan) as follows: 94°C for 1 min, 40 cycles of denaturation at 94°C for 10 s, annealing at 65°C for 30 s, and extension at 72°C for 20 s using a PCR thermal cycler (Takara). The primer pairs used for the PCR are listed in Table 1. The PCR products were electrophoresed on 2% agarose gel and made visible by Red Safe Acid Staining Solution (Chembio Ltd, Hertfordshire, UK).

**Table 1:** Primers for PCR used.

Target	Primer	Sequence (5’-3’)	Accession
hTERT	Forward	GCGGCCTGCTGCTGGATACC	NM_198253
	Reverse	GGCCGTGTCAGAGATGACGCG	
ACTB (for RT-PCR)	Forward	GCCAACCGCGAGAAGATGACCC	NM_001101
	Reverse	CTTGCGCTCAGGAGGAGCAATGATC	
CK12	Forward	CCAGGCGAGGTCAGCGTAGAA	NM_000223
	Reverse	CCTCCAGGTTGCTGATGAGC	
CK13	Forward	TAGCCCTGAGCGGGACTACA	* NM_153490
	Reverse	CAGGGCCAGCTCATTCTCAT	
MUC1	Forward	CCGGGATACCTACCATCCTATGAG	NM_001018016
	Reverse	GCTGCTGCCACCATTACCTG	
MUC4	Forward	GAAGACGTGCGCGATGTGA	NM_004532
	Reverse	CCTTGTAGCCATCGCATCTGAA	
MUC16	Forward	CTGCAGAACTTCACCCTGGACA	NM_024690
	Reverse	CCAAGCCGATGAGGATGACA	
ACTB (for real-time PCR)	Forward	TGGCACCCAGCACAATGAA	NM_001101
	Reverse	CTAAGTCATAGTCCGCCTAGAAGCA	

*Ramirez-Miranda A, Nakatsu MN, Zarei-Ghanavati S, Nguyen CV, Deng SX. Keratin 13 is a more specific marker of conjunctival epithelium than keratin 19. Mol Vis. 2011;17:1652–1661.

Real-time PCR was performed with the Power SYBR Green PCR Master Mix (Life Technologies), as follows: 95°C for 10 min, 40 cycles of denaturation at 95°C for 15 s, and annealing and extension at 60°C for 1 min using a Step One Plus^TM^ Real-Time PCR (Life Technologies). The primer pairs used for the real-time PCR are listed in Table 1. The Ct values were determined by the Step One Plus^TM^ Real-Time PCR software, and the amount of each mRNA was calculated relative to the amount of the mRNA of beta-actin (ACTB) in the same samples. Each run was completed with a melting curve analysis to confirm the specificity of the amplification and the absence of primer dimers.

### Immunofluorescence

The characteristics of the iHCjECs were evaluated by immunofluorescent staining for specific markers. The monoclonal antibodies that were used, their specificity, and dilution are presented in Table 2. For immunofluorescence staining of monolayer cultures, the iHCjECs (12th passage) were seeded at a density of 1.0 X 10⁴ cells/cm^2^ on culture slides (Matsunami, Tokyo, Japan) and cultured for 5 days in CnT-2D-Diff with or without Dox (1 μM) or 5% FBS-containing medium without Dox at 37°C with 5% CO_2._ The cells were fixed in 4% paraformaldehyde for 1 day and were then rinsed in phosphate-buffered saline (PBS).

**Table 2:** Characteristics of antibodies used in the characterization of iHCjECs.

Antibody	Specificity	Dilution	Species	Source
CK13(AE8)	Conjunctival epithelium	1:400	Mouse	Santa Cruz Biotechnology Inc
CK19	Conjunctival epithelium	1:100	Mouse	Dako North America Inc, Carpinteria, CA
MUC5AC	Mucosecretory cells	1:500	Mouse	Gene Tex
KL-6	a sialyated sugar moiety of MUC1	1:500	Mouse	Sekisui Medical Co. Ltd., Tokyo, Japan

The human corneoscleral donor rims that consisted of peripheral and limbal corneas and a small section of the bulbar conjunctiva were obtained from SightLife Surgical (Seattle, WA) Eye Bank. This tissue was what was left after removal of the central corneal button for transplantation. The normal human conjunctival tissues and the organotypic culture of iHCjECs were fixed in 4% paraformaldehyde and embedded in paraffin. The paraffin-embedded samples were cut into 5 μm sections and subjected to antigen retrieval by covering the sections with 10 M sodium citrate buffer (pH 6.0) and heating in a microwave oven for 16 min at 500 W. The slides were then cooled to room temperature and rinsed with PBS.

The slides were then incubated in blocking buffer, 0.1% Triton X-100, 0.01% sodium azide, 2% normal horse serum, 2% bovine serum albumin (BSA) in PBS, for 1 hour at room temperature. The slides were incubated with primary antibodies overnight at 4°C (Table 2). For the staining of CK12, CK13, CK19, and MUC5AC, the slides were covered with a fluorescein-conjugated secondary antibody for mouse IgG (1:500, VECTOR Laboratories, Burlingam, CA) solution for 1 hour at room temperature. The slides were counterstained with DAPI. The fluorescent images were photographed with a fluorescence microscope (BZ-9000; KEYENCE, Osaka, Japan). For the staining of CK13 and KL-6, the immunoreactivity to the primary antibodies was made visible with a DAB staining kit (VECTOR Laboratories), and the sections were counterstained with hematoxylin, dehydrated, and mounted with mounting medium. The sections were photographed with an Olympus BX50 microscope (Olympus, Tokyo, Japan) coupled with an Axio Cam lCc5 digital camera (Zeiss, Tokyo, Japan).

## Results

### 1. Expression of SV40LT antigen and hTERT gene in iHCjECs

Western blotting was performed to determine whether the SV40LT antigen was expressed in the iHCjECs cultured with and without Dox. The results showed that the SV40LT antigen was expressed in the cells cultured in a medium containing Dox, and the expression gradually decreased at 24 h, 48 h, and 72 h after replacing the medium with one without Dox. No expression of SV40LT antigen was observed in the cells cultured for 72 h in the medium without Dox (Fig 2A). On the other hand, the expression of the *hTERT* gene in iHCjECs was observed in the cultured cells either with or without Dox (Fig 2B). These results indicated that the Dox-dependent inducible SV40LT antigen was successfully introduced into the iHCjECs, and Dox was required to express the SV40LT antigen in iHCjECs while the expression of the *hTERT* gene was independent of Dox.

### 2. Cell morphology and growth curve of iHCjECs

**Fig 3:**
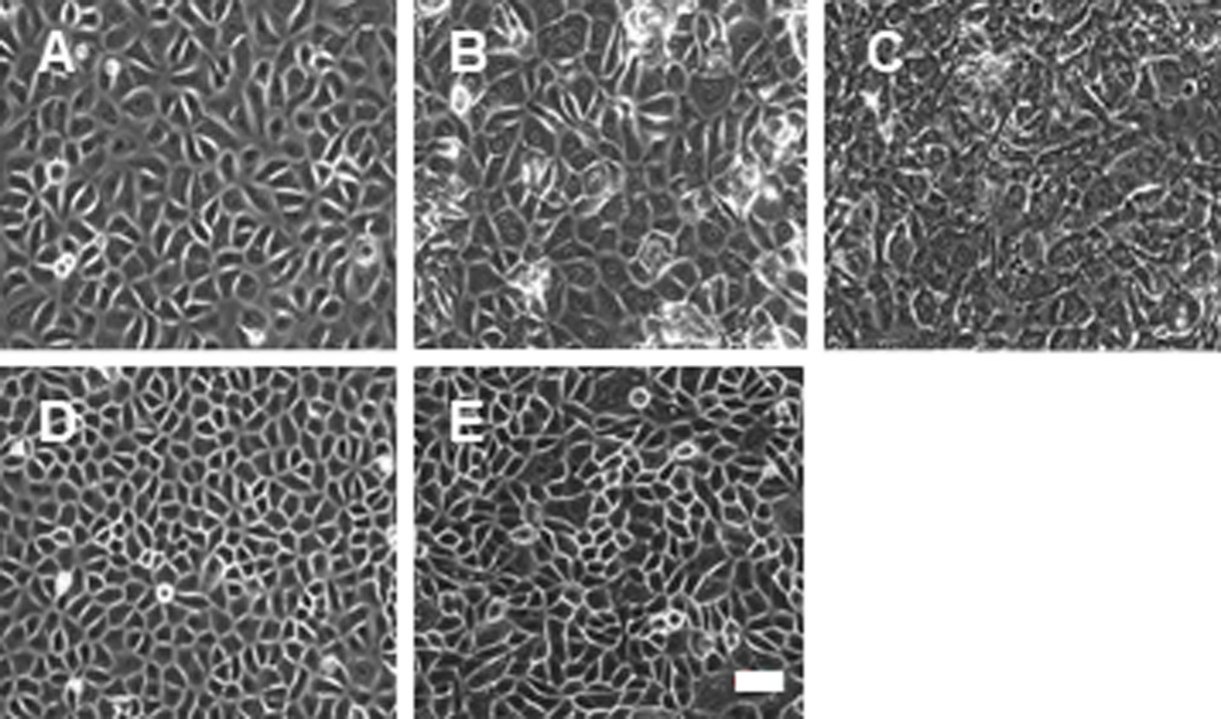
Photomicrographs of cultures of iHCjECs and primary culture cells. iHCjECs (12th passage) cultured with Dox (A) or without Dox (B) or without Dox containing 5%FBS (C) for 5 days. The iHCjECs cultured without Dox or without Dox containing 5%FBS for 5 days also maintained an epithelial-type morphology. Primary culture cells (E) and iHCjECs of later passages (20 passages) cultured with Dox (D) also showed cobblestone-like appearance. Bar = 50 μm.

The iHCjECs continued to replicate for at least 20 passages in the medium containing 1 μM Dox, and they appeared cobblestone-like under a microscope (Fig 3A and 3D). The morphology of the primary culture of HCjECs did not change significantly at each passage (Fig 3E). The iHCjECs cultured in Dox-free medium or in 5% FBS containing Dox-free medium for 5 days also maintained an epithelial-type morphology (Fig 3B and 3C).

**Fig 4:**
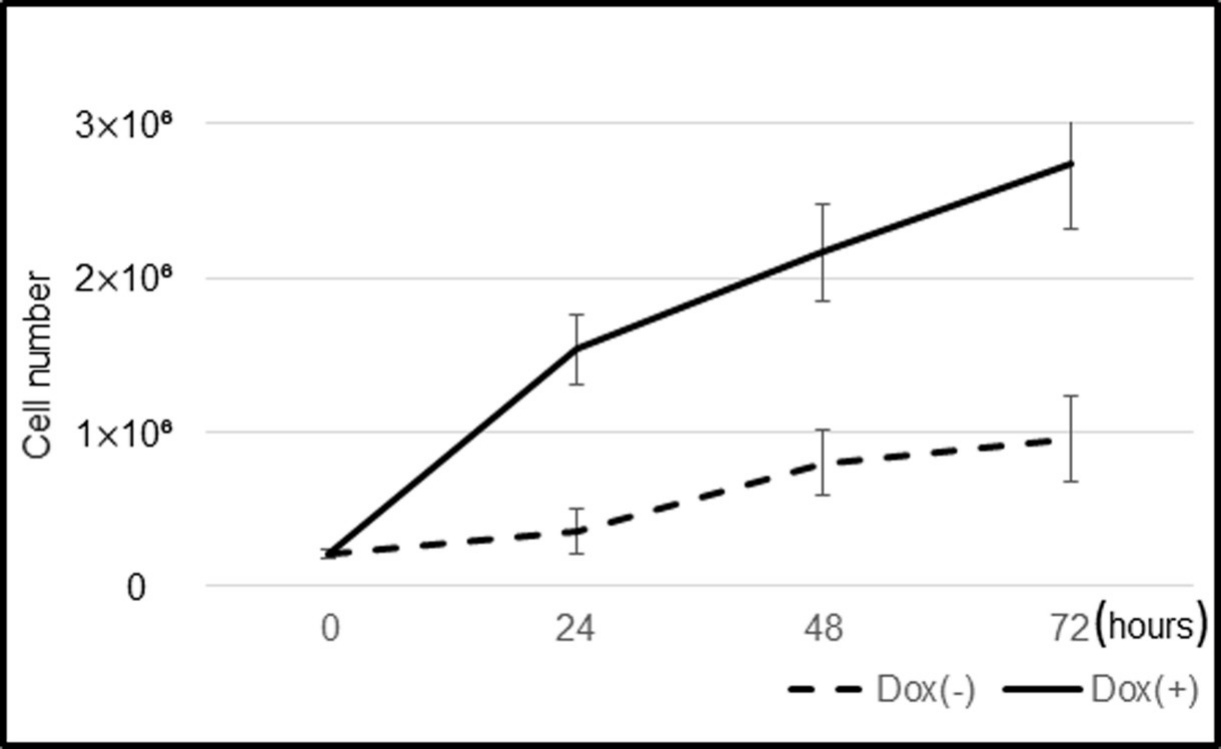
Growth curve of iHCjEC. The population doubling time of iHCjECs was 10.8 hours with Dox and 16 hours without Dox. The iHCjECs cultured without Dox grew less vigorously and could not be subcultured.

The population doubling time of the iHCjECs was 10.8 hours with Dox, and it was 16 hours without Dox. (Fig 4) The iHCjECs cultured with Dox had a high ability to proliferate, and they replicated less actively and could not be subcultured in media without Dox.

### 3. Expression of cytokeratin and mucins (MUC1, 4, 16, 5AC)

**Fig 5:**
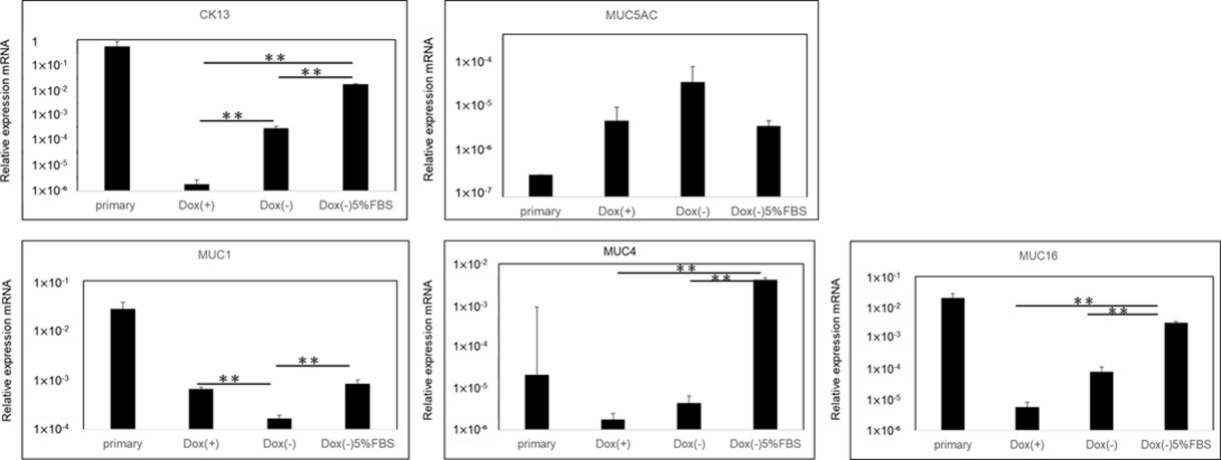
Expression of conjunctival epithelial cell-specific cytokeratin 13 (CK13) and mucins (MUC1, 4, 16, 5AC) determined by real-time PCR. The level of the expression of the mRNA of CK13 was higher in iHCjECs cultured without Dox than with Dox. ** indicates p-value < 0.05 as determined by Steel-Dwass test. The cultured iHCjECs expressed the mRNAs of the membrane-associated mucins, MUC1, MUC4, and MUC16 although their levels were lower. However, the expression of the mRNA of MUC1, MUC4, and MUC16 in cultures without Dox including 5% FBS remained at a high level close to that of the native cell cultures. ** indicates p-value < 0.05 as determined by Steel-Dwass test. The iHCjECs expressed the mRNAs of the secreted mucins, MUC5AC regardless of the presence or absence of Dox although their levels were low. There was no significant difference between them.

The iHCjECs expressed cytokeratin (CK) 13 which is known as a lineage-specific differentiation marker for human conjunctival epithelium [16]. The level of the mRNA of CK13 was higher in iHCjECs cultured without Dox than with Dox. (Fig 5) The corneal epithelial differentiation marker CK12 was not expressed in iHCjECs regardless of the presence or absence of Dox. (S1 Fig)

As in native tissues, the iHCjECs expressed the mRNAs of the membrane-associated mucins, MUC1, MUC4, and MUC16, although their levels were lower. On the other hand, the expressions of the mRNA of MUC1, MUC4, and MUC16 in cultures without Dox including 5% FBS were at high levels that were close to that of the primary cell cultures. The iHCjECs expressed the mRNAs of the secreted mucins, MUC5AC regardless of the presence or absence of Dox, although their levels were low. (Fig 5)

**Fig 6:**
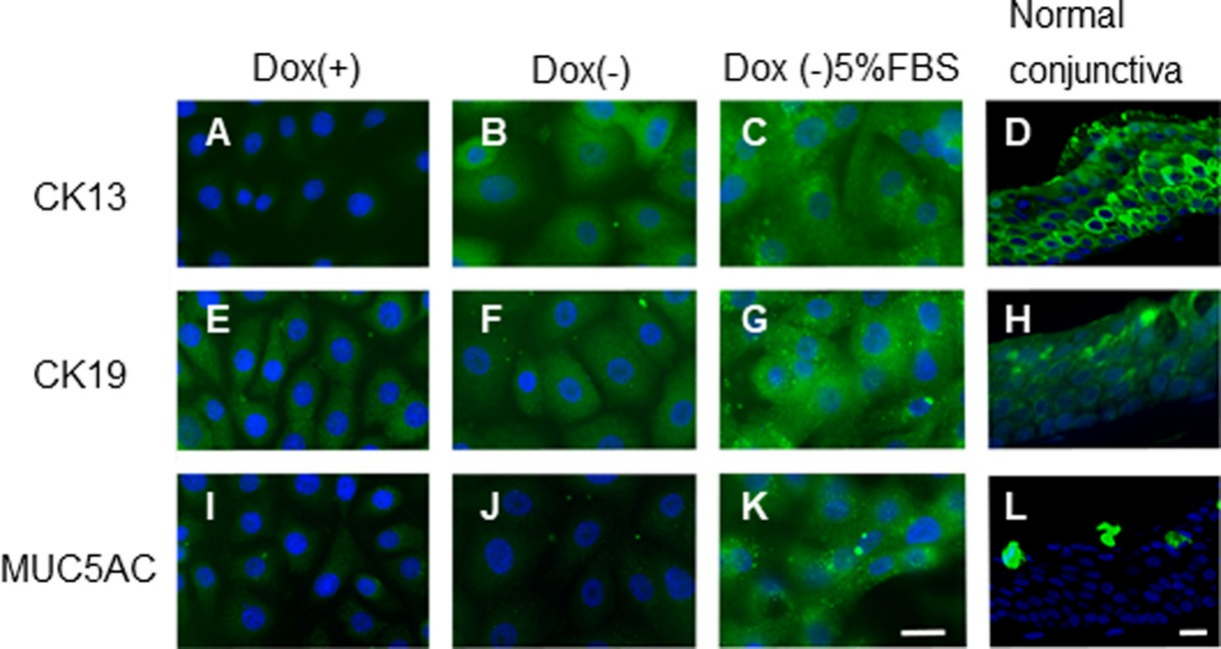
Immunofluorescence demonstrating expression of cytokeratin13, 19, 12 and MUC5AC in iHCjECs cultured on type I collagen-coated culture inserts. The density of CK13-positive cells was very low in the presence of Dox(A), but cells were present on the 5th day after the Dox-free media. (B) After changing to a medium without Dox supplemented with 5% FBS, the number of CK13-positive cells was increased on the 5th day. (C) Cytokeratin 19, which forms part of the cytoskeleton of conjunctiva epithelial cells, was positive in all cultures with or without Dox. (E, F, G) In addition, immunostaining of secreted mucin MUC5AC showed low positively-stained cells regardless of the presence or absence of Dox (I, J), however after exchanging to a medium supplemented with 5% FBS in a medium without Dox, partly stained cells were observed on day 5. (K). Bar = 20 μm.

Immunofluorescence staining showed that few CK13-positive cells were present in the cultures with Dox (Fig 6A), whereas the CK13-positive cells were found on the 5th day after replacing the Dox-containing cultures with a Dox-free medium either with or without 5% FBS (Fig 6B and 6C). CK19, which forms part of the cytoskeleton of conjunctiva epithelial cells, was expressed in the iHCjECs either with or without Dox (Fig 6E, 6F and 6G). In addition, immunostaining for secreted mucin MUC5AC showed few positively-stained cells regardless of the presence or absence of Dox (Fig 6I and 6J), however after exchanging to a medium supplemented with 5% FBS in a medium without Dox, positive stained cells were observed on day 5 (Fig 6K).

### 4. Organotypic culture of iHCjECs with conjunctival fibroblast-embedded collagen gel

**Fig 7:**
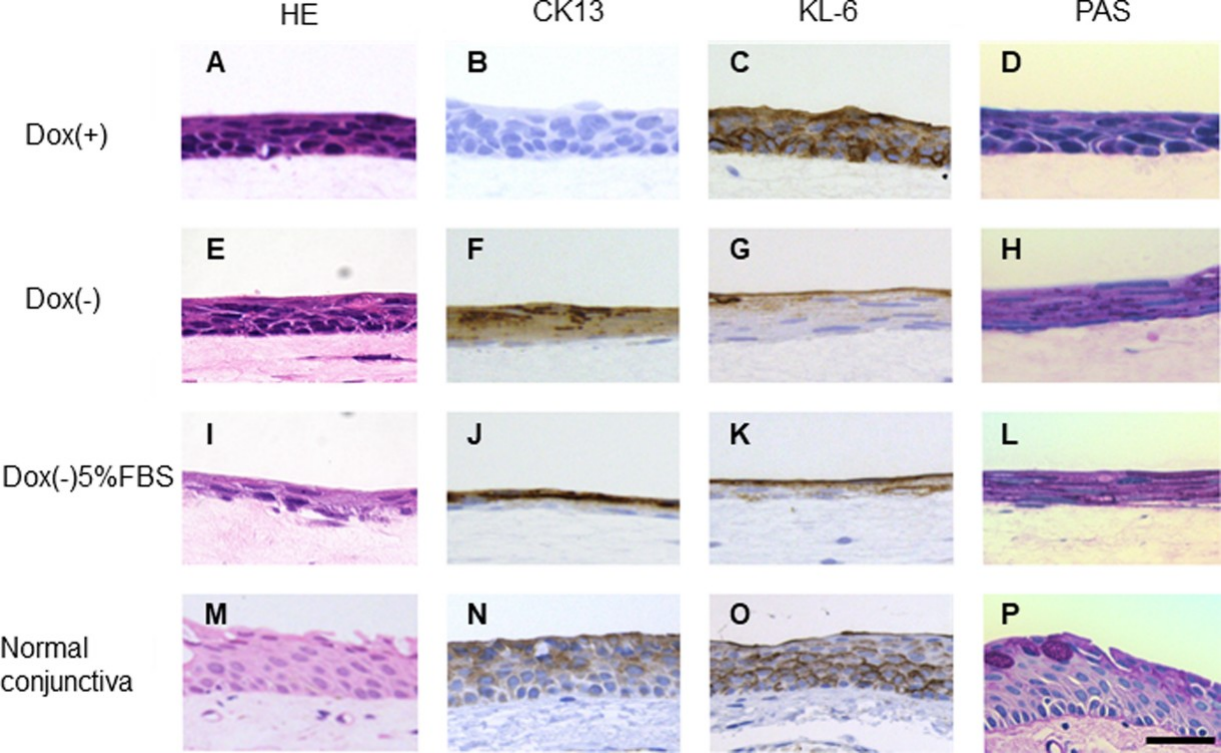
Investigation of cultures of rabbit conjunctival fibroblast-containing collagen gel. In three-dimensional cultures, the conjunctival epithelial cells stratified under all culture conditions either with or without Dox. (A, E, I) Immunostaining showed the expression of CK13 in the absence of Dox (F) and in the absence of Dox containing 5% FBS. (J) However, no positive cells were observed in the presence of Dox. (B) In addition, the expression of KL-6 was observed in the apical cells in the absence of Dox either with or without 5% FBS and in all cell layers in the cultured cells in the presence of Dox. (C, G, K) PAS staining showed no fully differentiated goblet cell in iHCjECs regardless of the presence or absence of Dox. (D, H, L). Bar = 50 μm.

The iHCjECs cultured on RCjFs-embedded collagen gel stratified either with or without Dox (Fig 7A, 7E and 7I), but the stratification was not observed when the iHCjECs were cultured in Dox-free medium without collagen gel. Immunostaining for CK13 in the organotypic culture showed no CK13-positive cells in the cultured cells in the presence of Dox. (Fig 7B) The suprabasal and superficial cells of the cultured cells expressed CK13 in the absence of Dox either with or without 5% FBS. (Fig 7F and 7J) In addition, the expression of MUC1 was detected by immunostaining of KL-6 which recognizes a carbohydrate epitope of MUC1 [17]. The expression of KL-6 was observed in the apical cells in the absence of Dox either with or without 5% FBS and in all cell layers in the cultured cells in the presence of Dox (Fig 7C, 7G and 7K). For PAS staining, few positive staining was detected in the presence with Dox. Although positive staining was detected in the absence of Dox either with or without 5% FBS, no fully differentiated goblet cell like appearance was detected in iHCjECs regardless of the presence or absence of Dox. (Fig 7D, 7H and 7L)

## Discussion

We have developed an iHCjEC line which was able to control the expression of the SV40 LT-antigen with a Tet-regulated gene-expression system. Thus, this control of the expression of SV40 LT-antigen led to iHCjECs with high proliferation rates in the presence of Dox, and they were also capable of differentiating into a conjunctival epithelial phenotype in the absence of Dox.

In earlier studies, several conjunctival cell lines [8,9,18] or cultured primary cells [3,4,5,19,20] have been reported, and they were used for various experimental studies. The differentiation of conjunctival epithelial cells has been demonstrated in three-dimensional cultures including fibroblasts using primary cultured cells [20], and they have been used for studies such as an analysis of inflammation-related factors and receptors [21,22]. However, there are some difficulties in conducting these experiments with the cultured primary conjunctival epithelial cells because it is well known that cultured primary conjunctival epithelial cells have poor proliferative ability [3]. Thus, it has been difficult to supply cultured primary conjunctival epithelial cells resulting in the shortage of the cells for experimentations.

To overcome the poor proliferative ability of primary cells, numerous cell lines have been developed for research purposes. For conjunctival epithelial cells, several immortalized human conjunctival epithelial cell lines have been reported. The so-called Chang conjunctival cell line [6] is listed as conjunctival in origin, however it is commonly acknowledged that it has a fibroblastic phenotype and Hela cell contamination. Thus, the Chang conjunctival cell line may express other cell types and may not represent a typical conjunctival epithelial cell phenotype.

Two other immortalized human conjunctival epithelial cell lines, a spontaneously immortalized cell line, called IOBA-NHC, and the hTERT, p53 and p16 abrogated immortalized cell line have been reported [8,18]. Some of the conjunctival epithelial cell phenotype was maintained in these two cell lines.

These cultured primary cells and the immortalized cell lines have some advantages and disadvantages for in vitro experiments. Therefore, a cell line that has both proliferative and differentiation capabilities is needed for in vitro experiments.

We have established a human conjunctival epithelial cell line that was able to control the expression of the introduced SV40LT antigen by a Tet-regulated gene-expression system. In the Tet-regulated gene-expression systems, a transcriptional regulation of the targeted gene relies on the activity of a trans-regulatory protein that can be activated or repressed by Dox. Thus, in our iHCjEC line, SV40LT is expressed continuously resulting in a high proliferative ability in the presence of Dox, while SV40LT is not expressed without Dox resulting in a differentiation ability. In addition, it is known that hTERT can contribute to the immortalization of cells and has less effect on the cellular differentiation potential [10,11]. Therefore, the *hTERT* gene was also introduced in a manner that is continuously expressed regardless of the presence of Dox.

Our iHCjEC line had high proliferative ability in the presence of Dox and was continuously subcultured for at least 20 passages. The expression of the mRNA of CK13 in iHCjECs showed that the expression level of CK13 decreased in the presence of Dox, and its expression increased in the absence of Dox. In the immunostaining of CK13, only weak staining of CK13 was observed in the presence of Dox, whereas CK13 staining was confirmed in most of the cells in the absence of Dox. Positive immunostaining of CK19 was observed regardless of the presence or absence of Dox.

It is known that CK13 and CK19 are expressed in normal conjunctival epithelial cells. However, CK19 is also expressed in some corneal epithelial cells, and thus CK13 has been reported to be a more specific marker for conjunctival epithelial cells [16]. The immunostaining of CK13 in normal human conjunctival epithelial cells is strong in the superficial and suprabasal cells and weak in the basal cells. These results suggest that CK13 may be expressed in more differentiated conjunctival epithelial cells and reflect the characteristics of mature conjunctival epithelial cells. Therefore, differentiation into more mature conjunctival epithelial cells may be promoted by suppressing the expression of SV40LT antigen in iHCjECs. As CK19 was expressed regardless of the presence or absence of Dox, it is likely that CK19 may be expressed in more undifferentiated cells or without an association with differentiation. However, the relationship between cytokeratin expressions and differentiation has not been determined, and further studies on the differentiation of conjunctival epithelial cells will be necessary.

The presence of mucins on the epithelial cell surface has been reported to be necessary for hydration and lubrication due to their heavy O-glycosylated regions [1]. In addition, the mucins play additional roles as barrier functions, cell growth, and differentiation, cell-cell and cell-matrix interactions, and signal transduction [23].

Our results showed that the mRNAs of MUC1, 4, and 16 were expressed in the iHCjECs similar to that of cultured primary human conjunctival cells although their expression levels were lower than that of the cultured primary cells.

When compared to the two other immortalized human conjunctival cell lines, the presence of mucins on the epithelial cell surface in the iHCjECs did not show obvious differences. This indicated that these three cell lines including our iHCjECs maintained some of the conjunctival epithelial cell phenotype. An important factor in studying conjunctival epithelial cells has been to identify goblet cells in the cultured cells. In primary cell culture systems, goblet cells-like MUC5AC positive cells have been detected [4,5]. However, no MUC5AC was detected by PCR in the IOBA-NHC [18]. Although a weak expression of MUC5AC was detected, fully differentiated goblet cells were not found in the hTERT, p53, and p16 abrogated immortalized cell line [8]. The real time PCR results showed the expression of MUC5AC by the iHCjECs regardless of the presence or absence of Dox. Although real time PCR results may not reflect the protein expression, the immunostaining results showed a faint expression of MUC5AC in the media with Dox (-) and relatively clear expression of MUC5AC in the media with Dox (-) and 5% FBS. In the hTERT, p53, and p16 abrogated immortalized cell lines, the authors reported that the goblet cells had been already lost when hTERT was transfected into the original conjunctival cells at four passages [8,24]. Thus, the population of the MUC5AC-positive cells was low in their cell lines. Contrary to the hTERT, p53, and p16 abrogated immortalized cell lines, the iHCjECs were transfected at the primary culture without subculturing. Thus, compared to other cell lines, the iHCjECs may maintain a potential to differentiate into goblet cells. Although MUC5AC expressions was detected in the iHCjECs, the cells did not fully differentiate into goblet cells. The molecular mechanisms of goblet cell differentiation in mucosal tissues have been intensively studied using genetic modified mice [25–28]. However, little has been clarified in human tissues. Therefore, further investigations will be necessary to determine the molecular mechanisms controlling the full differentiation of goblet cells, and iHCjECs can be a good candidate cell line to study the mechanisms of goblet cell differentiation in the human conjunctiva.

The KL-6 epitope is a sialylated sugar moiety of MUC1, and we examined its site of expression on the ocular surface epithelium. The KL-6 epitope is downregulated in the conjunctiva of severe dry eyes which may be explained in part by the malfunction of conjunctival epithelial cells [17]. In three-dimensional organotipic cultures, KL-6 antibody was detected in the stratified iHCjECs. These results indicated that iHCjECs could be useful in testing mucus secretagogues in vitro.

We have demonstrated that our iHCjECs have high proliferative ability as well as differentiation potency, however there are some limitations. Although iHCjECs expressed MUC5AC by PCR and immunohistochemically, we could not demonstrate the presence of goblet cells morphologically. It has not been fully determined how the differentiated status of iHCjECs cultured without Dox mimic the primary cultured human conjunctival epithelial cells or conjunctival epithelia in vivo because we tested only a few culture conditions in the present investigations. Future experiments are needed to clarify the complete properties of the iHCjECs. However, these iHCjECs can be used for in vitro investigations of conjunctival epithelia because their high proliferation ability will allow the investigators to use iHCjECs any time without shortage of cultured cells. The differentiation potency of iHCjECs will allow investigators to study the characteristics of human conjunctival epithelial cells, although further investigations will be necessary on the properties of the iHCjECs. In addition, both proliferation and differentiation potential may be useful and convenient in the experimental design.

In conclusion, we have developed an immortalized conjunctival epithelial cell line, iHCjEC, which was able to control the expression of SV40 LT-antigen with Tet-regulated gene-expression system. Because iHCjECs have high proliferative ability as well as differentiation potency, iHCjECs may be a useful experimental cell line for experimental studies of the properties of conjunctival epithelial cells as well as for physiopathological or toxicological studies.

## Supporting information

S1 Fig PCR of CK12 expression in iHCjECs cultured with Dox or without Dox. The corneal epithelial differentiation marker CK12 was not expressed in iHCjECs regardless of the presence or absence of Dox. HCEs: Human corneal epithelial cells were scraped from human corneas obtained from SightLife Surgical Eye Bank (Seattle, WA).
(TIF)
