# Supplementary figures and images for: Characterization of doxycycline-dependent inducible Simian Virus 40 large T antigen immortalized human conjunctival epithelial cell line
Source: PLoS One. 2019 Sep 11;14(9):e0222454. doi: 10.1371/journal.pone.0222454 (PMC6738650; doi:10.1371/journal.pone.0222454)

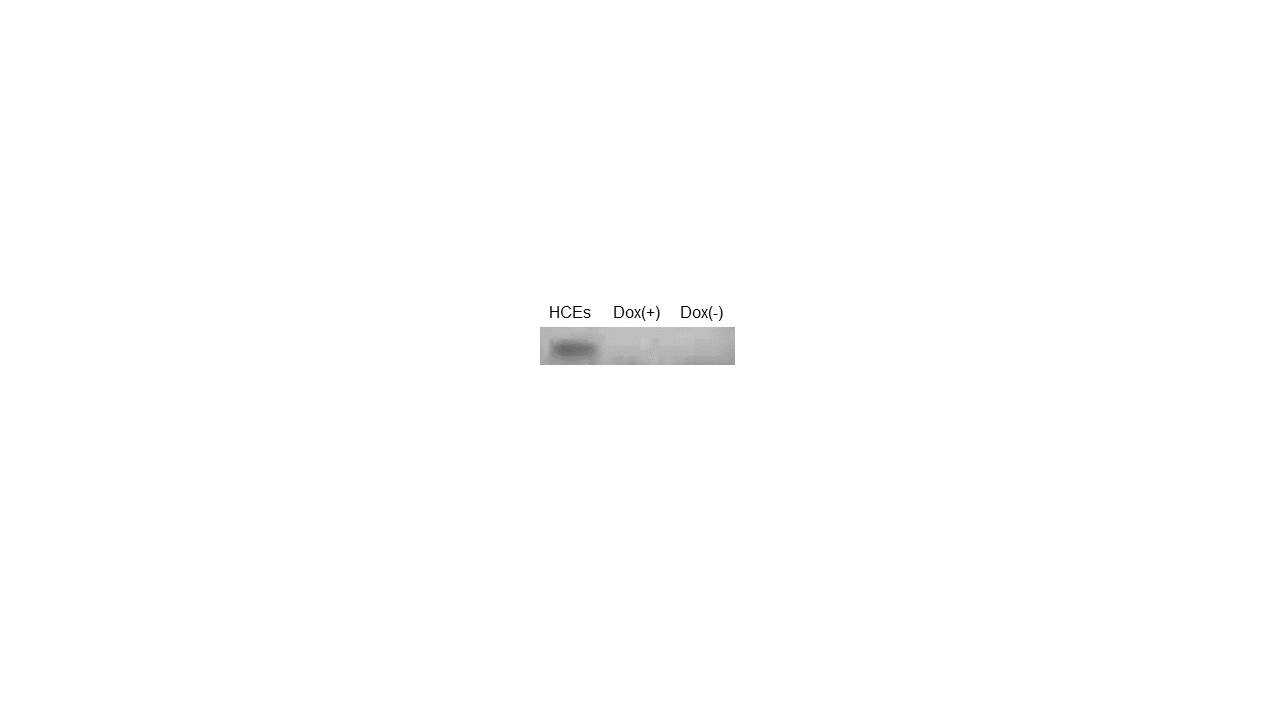

Supplement: S1 Fig — The corneal epithelial differentiation marker CK12 was not expressed in iHCjECs regardless of the presence or absence of Dox. HCEs: Human corneal epithelial cells were scraped from human corneas obtained from SightLife Surgical Eye Bank (Seattle, WA). (TIF) [file pone.0222454.s001.tif]
